# Sudden-Onset Blindness in a Child With Hypertensive Emergency: Unmasking of Chronic Kidney Disease

**DOI:** 10.7759/cureus.29233

**Published:** 2022-09-16

**Authors:** Koyel Chakraborty, Deepanjan Bhattacharya, Poonam Kanwar

**Affiliations:** 1 Ophthalmology, Regional Institute of Ophthalmology, Medical College and Hospital, Kolkata, IND; 2 Pediatrics, Postgraduate Institute of Medical Education and Research, Chandigarh, IND

**Keywords:** fundus, hypertension, chronic kidney disease, macular star, hypertensive retinopathy

## Abstract

A 10-year-old male presented with sudden-onset diminution of vision in both eyes. On systemic examination, he had severe hypertension, no pulse deficit, short stature, and no other focal neurological deficit. Dilated fundoscopy showed bilateral grade 4 hypertensive retinopathy with macular star formation. Detailed laboratory investigations revealed a stage 5 chronic kidney disease (CKD). We present this case to highlight this rare ocular manifestation of CKD in pediatric age group.

## Introduction

We report a 10-year-old male who presented with sudden-onset blindness secondary to hypertensive retinopathy, resulting from undetected chronic kidney disease.

## Case presentation

**Figure 1:**
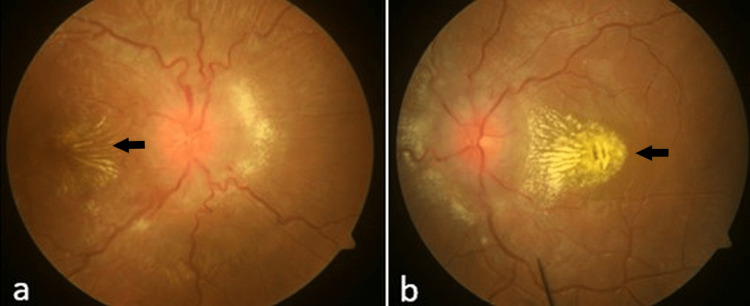
Fundus photograph showing grade 4 hypertensive retinopathy with macular star (black arrows) in the right eye (a) and the left eye (b)

A 10-year-old boy presented with sudden-onset headache, vomiting, and diminution of vision in both eyes for the last six hours. It was not associated with loss of consciousness or seizures. There was no history of similar illness in the past or significant medical or surgical history. On general examination, he had a blood pressure of 220/110 mm Hg, with no discordance between the upper and lower limbs and no pulse deficit. He was started on labetalol infusion and oral amlodipine (10 mg/day). Detailed physical examination revealed short stature (height: 110 cm) and moderate pallor, without any focal neurological deficit. Visual acuity was 6/60 (Snellen) in both eyes. Fundus examination showed bilateral grade 4 hypertensive retinopathy with macular star formation (Figure 1).

Investigations revealed normocytic normochromic anemia (hemoglobin: 72 gm/l) with deranged renal function (blood urea nitrogen: 17.01 mmol/l {normal: 14.3-17.8} and creatinine: 0.584 mmol/l {normal: 0.04-0.13}) and normal anion gap metabolic acidosis (pH: 7.217 and HCO3: 12 meq/l {normal: 24-28}), without any evidence of dyselectrolytemia. Urinalysis showed 1+ albuminuria with 24-hour albumin being 10 mg per m^2^ body surface area and no abnormality on microscopy. Renal ultrasound showed bilateral shrunken kidneys with loss of corticomedullary differentiation and no evidence of hydronephrosis. Electrocardiogram did not show any abnormality, and echocardiogram showed good biventricular function with left ventricular hypertrophy and no evidence of coarctation.

His blood pressure was controlled over the next 48 hours, and he was switched to oral labetalol and amlodipine. 25-Hyrdroxyvitamin (OH) D3 was found to be low (5 ng/ml), and parathormone was elevated (200 pg/ml) with borderline calcium (7.8 mg/dl) and phosphate (6.7 mg/dl). Iron studies showed low serum ferritin (10 ng/ml). He was given intravenous iron with oral vitamin D and calcium supplementation, along with oral sodium bicarbonate for metabolic acidosis. At the time of discharge, his vision improved to 6/9 (Snellen) in both eyes. Repeat fundus examination after three months showed resolution of the macular deposits and regression of hypertensive changes. Weekly erythropoietin therapy was started on follow-up, and his blood pressure was found to be between the 50th and 75th centile.

## Discussion

In children, any blood pressure more than 99th percentile for age and height, with features of end-organ damage (hypertensive encephalopathy, stroke, retinal hemorrhage or ischemia, myocardial ischemia, acute left ventricular dysfunction, acute pulmonary edema, aortic dissection, or acute renal failure), is termed as hypertensive emergency [1]. Its prevalence in the pediatric population ranges from 1% to 19%, with 75%-80% due to secondary causes [2]. Renal and renovascular causes are the commonest etiologies, followed by coarctation of the aorta, pheochromocytoma, and paragangliomas. Hypertensive emergencies can damage vessel walls leading to endothelial injury, fibrinoid necrosis, and the activation of the coagulation cascade. Management includes reduction of blood pressure to below 90th percentile for age and sex over a period of 24-72 hours, avoiding sudden drastic reductions. It encompasses the use of parenteral antihypertensives such as labetalol and sodium nitroprusside along with oral therapy with calcium channel blockers [3].

Hypertension in children has a prevalence of 4% worldwide but is grossly under-recognized [4]. It is a marker of hypertension and metabolic syndrome in adulthood and is associated with higher degrees of end-organ damage [5]. Moin et al. examined 62,982 apparently normal children and found that 10% had abnormal blood pressure recordings, with guideline-adherent follow-up seen in only 13% [6]. Studies from India have shown the prevalence of hypertension among children being 0.4%-11% with poor rate of detection. Higher prevalence was seen among those who were overweight or from an urban background [7]. Hypertensive retinopathy has been reported to occur in 8%-18% of children with severe hypertension, which is much less compared to adults (70%-80%) [8]. Higher prevalence is seen in those with renal parenchymal and renovascular disease [9].

The pathogenesis includes vasoconstriction of arterioles followed by sclerosis and exudates resulting from the disruption of the blood-retina barrier. Vision loss is due to secondary optic atrophy after prolonged papilledema or retinal pigmentary changes after exudative retinal detachment. Strict control of hypertension is the only effective treatment, with high mortality among those who remain untreated.

Retinopathy due to anemia was an important differential diagnosis in our case. However, it is usually asymptomatic, with findings varying from retinal hemorrhages, cotton wool spots, venous tortuosity, and occasionally white-centered hemorrhages called Roth spots [10]. The dysregulation of the coagulation cascade can lead to the occlusion of retinal vessels, with contributions from reactive thrombocytosis and hypoxia-induced endothelial injury [11]. In our index case, anemia was not severe, and the patient had a visual loss with markedly elevated blood pressure.

Chronic kidney disease in children is an under-recognized entity with a reported prevalence of 50-60 per million, with a high prevalence of short stature and hypertension with end-organ damage [12]. Kanitkar et al. reported the scarcity of data about pediatric chronic kidney disease from India, with long latencies before diagnosis and a higher degree of progressive disease at the time of diagnosis [13].

Our index case demonstrates the delayed presentation of children with chronic kidney disease and higher degrees of end-organ damage. Hypertensive retinopathy with macular star formation represents the advanced degree of end-organ damage seen and highlights the need for early diagnosis of this group of patients.

## Conclusions

Chronic kidney disease in children is often missed due to a long asymptomatic period and can have initial manifestation as a hypertensive emergency. Prompt diagnosis is important, with early initiation of appropriate management strategies.

## References

[1] Majdalani MN (2010). Management of hypertensive emergencies in children. J Med Liban.

[2] Chandar J, Zilleruelo G (2012). Hypertensive crisis in children. Pediatr Nephrol.

[3] Raina R, Mahajan Z, Sharma A (2020). Hypertensive crisis in pediatric patients: an overview. Front Pediatr.

[4] Song P, Zhang Y, Yu J, Zha M, Zhu Y, Rahimi K, Rudan I (2019). Global prevalence of hypertension in children: a systematic review and meta-analysis. JAMA Pediatr.

[5] Sun SS, Grave GD, Siervogel RM, Pickoff AA, Arslanian SS, Daniels SR (2007). Systolic blood pressure in childhood predicts hypertension and metabolic syndrome later in life. Pediatrics.

[6] Moin A, Mohanty N, Tedla YG, Carroll AJ, Padilla R, Langman CB, Smith JD (2021). Under-recognition of pediatric hypertension diagnosis: examination of 1 year of visits to community health centers. J Clin Hypertens (Greenwich).

[7] Uddaraju A, Ram CV (2013). Hypertension in children, not a "small" problem. Indian Heart J.

[8] Williams KM, Shah AN, Morrison D, Sinha MD (2013). Hypertensive retinopathy in severely hypertensive children: demographic, clinical, and ophthalmoscopic findings from a 30-year British cohort. J Pediatr Ophthalmol Strabismus.

[9] Foster BJ, Ali H, Mamber S, Polomeno RC, Mackie AS (2009). Prevalence and severity of hypertensive retinopathy in children. Clin Pediatr (Phila).

[10] Carraro MC, Rossetti L, Gerli GC (2001). Prevalence of retinopathy in patients with anemia or thrombocytopenia. Eur J Haematol.

[11] Leung KCP, Li KK (2020). Anaemia induced vision loss. BMJ.

[12] Harambat J, van Stralen KJ, Kim JJ, Tizard EJ (2012). Epidemiology of chronic kidney disease in children. Pediatr Nephrol.

[13] Kanitkar M (2009). Chronic kidney disease in children: an Indian perspective. Med J Armed Forces India.

